# Tuberculosis treatment outcomes and associated factors among patients treated at Bosaso TB Hospital, Bosaso, Somalia: A five-year retrospective study

**DOI:** 10.1371/journal.pone.0314693

**Published:** 2025-01-24

**Authors:** Saaid Said Jama, Mohamed Mohamud Abdi

**Affiliations:** 1 Faculty of Medicine, University of Health Sciences, Bosaso, Puntland, Somalia; 2 Administration Department, Ministry of Health (Puntland), Bosaso, Puntland, Somalia; Aga Khan University Hospital Nairobi, KENYA

## Abstract

**Introduction:**

Tuberculosis remains a major public health problem, primarily in low- and middle-income countries. Evaluating treatment outcomes and investigating factors associated with them are essential for the treatment and control of tuberculosis. Hence, this study aims to assess the TB treatment outcomes and associated factors in Bosaso, Puntland, Somalia.

**Methods:**

A 5-year facility-based retrospective study was conducted at Bosaso TB Hospital, from January 2018 to December 2022. A total of 2213 TB patients were included in this study. Demographic, clinical characteristics and treatment outcome data were gathered from the TB register using a structured checklist. Data were entered, cleaned, and analyzed using SPSS version 20. Descriptive statistics and binary logistic regression analysis were employed. A P-value of less than 0.05 was considered statistically significant.

**Results:**

The overall successful treatment rate was 88.5%. The TB treatment success rate over the last three years was comparable to the global target of the End TB strategy of ≥ 90% by 2025. Patients aged 21–40 years (AOR = 0.59, 95% Cl = 0.41–0.84, p = 0.004), 41–60 years (AOR = 0.37, 95% CI = 0.25–0.55, p < 0.001), and ≥ 61 years (AOR = 0.37, 95% CI = 0.22–0.64, p < 0.001) were less likely to achieve successful treatment outcomes. Being HIV-positive (AOR = 0.41, 95% Cl = 0.21–0.79, p = 0.008) was less likely to be associated with a successful treatment outcome.

**Conclusion:**

In this study, the TB treatment success rate over the last three years was comparable to the global target of the End-TB strategy of ≥ 90% by 2025. Therefore, we recommend strengthening the TB care system, conducting regular supportive supervision for TB facilities, implementing strategies to encourage drug adherence, strengthening mechanisms to minimize the anti-TB treatment default rate, improving counseling services, and giving more attention to the vulnerable age groups and HIV-positive patients.

## Introduction

Tuberculosis (TB) is considered the second leading cause of death from an infectious disease after coronavirus (COVID-19) and ranked above HIV/AIDS [1]. Globally, approximately 10.6 million people developed TB in 2022, and 1.3 million of them died [2]. Low- and middle-income nations, including Sub-Saharan Africa, contribute 94% of TB illnesses and deaths [3].

In Somalia, TB remains a major public health problem and a leading cause of morbidity and mortality. The rate of the disease burden has been relatively stable, with reports of 258 per 100,000 people in 2018 and 259 per 100,000 people in 2020 [4]. In 2022, TB had an estimated incidence of 43,000 (27000–63000) cases with a rate of 246 (155–359) per 100,000 people [5]. The incidence of TB in Somalia has decreased by 14% since 2010, due to an increased number of TB treatment facilities, improved referral mechanisms, community engagement, and the enhanced role of community health workers in detecting and referring suspected TB cases [6].

The treatment success rate is one of the most essential indicators for achieving the goals of the End TB strategy, which targets a rate of ≥ 90% by 2025 [7]. The WHO recommends that treatment outcome analysis be carried out annually at the national and health facility levels to improve disease management and control initiatives [8].

Several studies have reported TB treatment outcomes in Somalia [9, 10]. A cross-sectional study conducted in Galkayo, Puntland, found that patient body weight, diabetes, family size, delay in diagnosis, treatment frequency, smoking, patient category, and duration of treatment were associated with unfavorable treatment outcomes [11]. Furthermore, a study conducted in Mogadishu revealed that being married, educated, a new TB case, HIV negative, and having knowledge of TB were associated with successful treatment outcomes [12]. Monitoring TB treatment outcomes is critical to achieving the End TB strategy target rate of ≥ 90% of TB patients receiving treatment by 2025 [13]. However, to our knowledge, the treatment outcomes and associated factors have not yet been studied in Bosaso. Therefore, this study aims to assess the TB treatment outcomes and associated factors in Bosaso, Puntland, Somalia.

## Methods

### Study design and setting

A public health facility-based retrospective review of the TB treatment register was performed from January 2018 to December 2022 to assess TB treatment outcomes and associated factors among TB patients treated at Bosaso TB Hospital, the only center for diagnosing and treating tuberculosis in Bosaso, which is the capital of the northeastern Bari region and one of the most populated cities in Puntland State, Somalia. Bosaso TB Hospital is located in New Bosaso Village, in the southwestern part of Bosaso City. The facility started to function in 1996 as the Bosaso TB Center, and in 2020 it was promoted to a hospital. The hospital screens around 2100–2500 individuals for TB and treats 450–500 TB patients annually.

### Study population and sample size

All TB patients who were registered during the period of the study were included in the study. A total of 2271 patients were registered. We excluded 51 patients who were transferred to other facility as multi-drug resistant cases and 7 patients who had incomplete data on treatment outcomes. We analyzed data from 2213 (97.45%) patients with complete treatment outcomes.

### Study procedure

We collected data on all eligible TB patients registered during the five-year study period between October 20 and December 31, 2023, using a structured checklist. A physician diagnosed TB patients and assessed their treatment outcomes at the hospital. Following the release of the diagnostic results and instructions for the patients to start treatment, the hospital data clerk recorded patient information in the TB register, including sex, age, TB type (pulmonary-PTB, extrapulmonary-EPTB), patient’s category, smear result, and HIV status. Additionally, the clerk recorded the treatment outcome for the patient at the end of their treatment period. Based on the recommendations of the Somali National Guidelines for TB, all presumed patients, including children who can produce sputum, underwent bacteriological evaluation of sputum using Gene Xpert MTB/RIF. The independent variables collected were age, sex, type of TB, patient category, smear results, HIV status, and year of treatment. The independent variables collected were age, sex, type of TB, patient category, smear results, HIV status, and year of treatment. The dependent variable was the status of the TB treatment, which was classified as either successful (cured or completed) or unsuccessful (treatment failure, death, lost to follow-up, or not evaluated).

### Data analysis

The data were entered into an Excel spreadsheet, the correctness of the data was checked, and any doubts were clarified with the hospital data clerk. Then the data were exported into SPSS (statistical packages for social sciences version 20) for analysis. Descriptive statistics were used to describe participants’ characteristics, treatment outcomes, and trends. Bi-variable and multivariable binary logistic regressions were applied to determine the factors associated with TB treatment outcomes. A crude and adjusted odds ratio (OR) with a corresponding 95% confidence interval (CI) were computed and reported. A p-value < 0.05 at a 95% confidence interval was considered statistically significant.

### Operational term definitions

The Somali National TB control program guidelines, WHO definitions and treatment framework for tuberculosis, and WHO treatment outcome definitions were used to define the operational terms [14, 15].

#### TB patient category

“New patients” are patients who have never received TB treatment or have taken anti-TB medication for less than 1 month.

“Previously treated patients” are patients who have received 1 month or more anti-TB medications in the past and are further categorized by the outcome of their most recent treatment course as relapse, treatment after failure, treatment after loss to follow-up, and other previously treated patients.

“Transfer-in” refers to a TB patient who received treatment in a tuberculosis unit, after being registered for treatment in another TB unit.

#### Treatment outcome variables

“Cured” is defined as a pulmonary TB patient who started treatment with bacteriologically confirmed TB and finished the treatment as advised by national policy with evidence of bacteriological response and no signs of failure.

“Treatment completed” refers to a patient who has completed the recommended treatment course according to national policy but did not meet the criteria for cure or treatment failure.

“Died” refers to a patient who died before the beginning of treatment or while receiving it.

“Lost to follow-up” refers to a patient who either doesn’t start therapy or halts their treatment for two consecutive months or longer.

“Not evaluated” refers to a patient for whom no treatment outcome was assigned. This applies to situations where the reporting unit is unsure of the treatment outcome or the patient has been transferred to another treatment facility.

### Ethical clearance

The study was conducted as per the Helsinki Declaration, and ethical approval was obtained from the Ethical Review Committee of the University of Health Sciences, Bosaso, Puntland, Somalia, with the need for written informed consent waived. A permission letter was also obtained from Bari regional office of the Ministry of Health of Puntland State, Somalia. No patient identifying information was collected. All data were kept confidential and used only for the intended purpose.

## Results

### Demographic and clinical characteristics of the TB patients

A total of 2213 TB patients were included in this study. Males accounted for 68.5% of the participants. About 34.9% of the patients were aged 20 years or less, 42.3% were in the age range of 21–40 years, and 16% and 6.7% of the study population were aged 41–60 years and ≥ 61 years, respectively. Most of the patients (63.9%) were diagnosed with pulmonary tuberculosis, and 67.1% of them were smear-positive. The majority of the participants (87%) were new TB cases. Among the participants, only 50 patients were HIV-positive (Table 1 and S1 Dataset).

**Table 1:** Demographic and clinical characteristics of the TB patients (n = 2213)*.

Variable	Frequency (N)	Percentage (%)
**Sex**		**n = 2213**
Male	1515	68.5
Female	698	31.5
**Age group**		**n = 2213**
≤ 20 years	773	34.9
21–40 years	937	42.3
41–60 years	355	16
≥ 61 years	148	6.7
**TB type**		**n = 2183**
Pulmonary	1396	63.9
Extra-pulmonary	787	36.1
**Pulmonary TB smear results**		**n = 1387**
Smear positive PTB	931	67.1
Smear negative PTB	456	32.9
**Patient category**		**n = 2176**
New	1894	87
Previously treated	113	5.2
Transfer in	169	7.8
**HIV status**		**n = 1988**
Positive	50	2.5
Negative	1938	97.5

*Missing: HIV status: 225 (10.17%) cases.

### Treatment outcomes among TB patients

Of the total TB cases included in this study, 805 (36.4%) were cured, 1154 (52.1%) completed their treatment regimen, 121 (5.5%) lost to follow-up, 84 (3.8%) died, 37 (1.7%) were not evaluated, and 12 (0.5%) were declared treatment failures. The overall rate of successful treatment outcomes (cured and completed) during the study period was 88.5%, as shown in Table 2 and S1 Dataset.

**Table 2:** Distribution of treatment outcomes among TB patients.

Variable	Frequency (n = 2213)	Percentage (%)
**Successful**	**1959**	**88.5**
Cured	805	36.4
Completed	1154	52.1
**Unsuccessful**	**254**	**11.5**
Lost to follow-up	121	5.5
Died	84	3.8
Not evaluated	37	1.7
Treatment failure	12	0.5

### Trends of successful treatment outcomes

**Fig 1:**
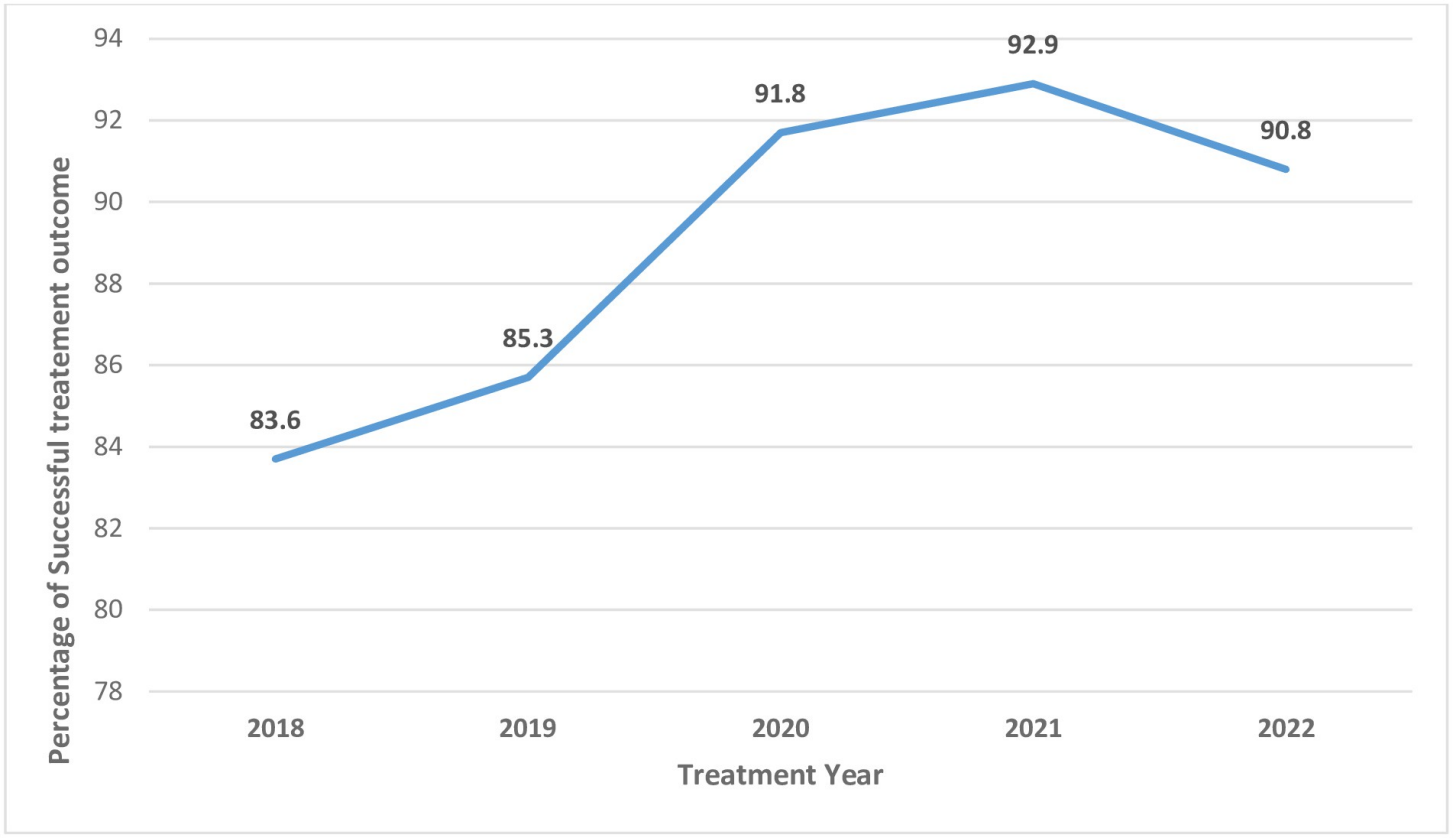
Trend of successful treatment outcomes of TB patients (n = 2213).

The trend of successful treatment outcomes showed a progressive yearly increase during the study period. The annual success rate reached above 90% targeted by the WHO End TB strategy from 2020 to 2022, as reported in Fig 1.

### Factors associated with tuberculosis treatment outcomes

The bivariate logistic regression analysis revealed a significant association between age and HIV status and the outcome of TB treatment. The multiple logistic regression indicated that patients aged 21–40 years were 41% less likely to have good treatment outcomes (AOR = 0.59, 95% Cl = 0.41–0.84, p = 0.004) compared to patients aged 20 years or younger. Also, patients aged 41–60 and 61 years and above were each 63% less likely to have successful treatment outcomes (AOR = 0.37, 95% CI = 0.25–0.55, p < 0.001) and (AOR = 0.37, 95% CI = 0.22–0.64, p < 0.001), respectively, compared to patients aged 20 years or younger. HIV-positive patients were 59% less likely to have successful treatment outcomes compared to HIV-negative patients (AOR = 0.41, 95% Cl = 0.21–0.79, p = 0.008), as shown in Table 3.

**Table 3:** Factors associated with tuberculosis treatment outcomes*.

Factor	Categories	Treatment Outcome		Bivariate analysis	*P*	Multivariate analysis	*P*
		S	U	COR (95% CI)		AOR (95% CI)	
**Sex**							
	Male	1341	174	Reference			
	Female	618	80	1.002 (0.757–1.328)	0.99		
**Age**							
	≤ 20 years	717	56	Reference			
	21–40 years	825	112	0.575 (0.411–0.805)	<0.001	0.59 (0.41–0.84)	0.004
	41–60 years	292	63	0.362 (0.246–0.532)		0.37 (0.25–0.55)	< 0.001
	≥61 years	125	23	0.424 (0.252–0.715)		0.37 (0.22–0.64)	< 0.001
**TB type**							
	PTB	1246	151	Reference			
	E-PTB	686	101	0.82 (0.63–1.08)	0.16		
**Smear results**							
	PTB+	839	93	Reference			
	PTB-	398	58	0.76 (0.537–1.078)	0.12		
**Patient category**							
	N	1679	215	Reference			
	P	95	18	0.68 (0.40–1.14)	0.28		
	T	152	17	1.15 (0.68–1.93)			
**HIV status**							
	Negative	1720	218	Reference			
	Positive	37	13	0.36 (0.19–0.69)	0.002	0.41(0.21–0.79)	0.008

*Abbreviations: S: successful, U-unsuccessful, PTB: Pulmonary TB, EPTB-extrapulmonary TB, N-new, P-previously treated patients, T-transfer-in. COR: crude odds ratio, AOR: adjusted odds ratio, CI- confidence interval.

## Discussion

This study assessed TB treatment outcomes and associated factors among TB patients treated at Bosaso TB Hospital from 2018–2022. We found an overall treatment success rate of 88.5%. This finding is higher than treatment success rates reported in Galkayo (85%), Mogadishu (81.8%), Southern Ethiopia (82.5%), and Uganda (55%) [11, 12, 16, 17]. On the contrary, our finding is slightly lower than successful treatment outcomes found in western Ethiopia (91.9%), Pakistan (94.91%), and Spain (89.4%) [18–20]. This discrepancy might be due to differences in the socio-economic status of the participants, access to health facilities, participants’ awareness, geographical setting, sample size, and study period.

This study found that patients’ age groups were significantly associated with TB treatment outcomes. Patients in age groups 21–40, 41–60, and ≥61 years were less likely to have successful treatment outcomes compared to patients aged 20 years or younger, in agreement with other studies conducted [17, 21]. Previous studies have suggested that increasing age may be associated with a higher chance of concomitant diseases, psychological deterioration, and the inability to travel to a healthcare facility, leading to a poor TB treatment outcome [22–24]. Furthermore, Khat use among adults is common in the study area, which could potentially lead to unsuccessful treatment outcomes as previous studies have found Khat use to be associated with a higher occurrence of TB and poor adherence to anti-TB medications [25, 26].

The current study showed that HIV-positive patients were 59% less likely to have successful TB treatment outcomes compared to their counterparts, consistent with previous reports [22, 27, 28]. The reasons for poor TB treatment outcomes among HIV-positive patients include depressed immune status, the presence of other comorbidities, and increased TB/HIV drug interaction and toxicity [29, 30]. Additionally, immunological studies have demonstrated that host responses against mycobacterium TB promote HIV replication. This, in turn, accelerates the progression of HIV and further reduces cellular immunity [31]. Another reason could be the potential negative impact of the stigma associated with TB/HIV diagnosis on the outcomes of TB treatment [32].

The findings of this study are important for shaping health policy, as they aid in enhancing TB services and devising interventions aimed at reducing TB incidence and its associated factors. For health practitioners, it highlights the factors associated with unsuccessful treatment outcomes, thereby necessitating improved TB patient counseling and monitoring in these cases.

This study has several limitations. It is based on the analysis of secondary data subject to missing information. To identify all factors, such as socioeconomic status, occupation, educational level, commodities, and others that might influence TB treatment outcomes, was impossible. There were limitations to using a TB register to determine outcomes, as not all records in it were completed, the diagnoses of patients who were smear-negative were often not available and would be required to assess validity and probability, and the clinical parameters needed to ascertain patient status were lacking.

## Conclusion

In this study, the overall TB success rate was 88.5%. The TB treatment success rate over the last three years was comparable to the global target of the End TB strategy of achieving ≥ 90% of patients receiving treatment by 2025. Increasing age and HIV-positive patients were significantly associated with unsuccessful treatment outcomes. We recommend reinforcing the TB care system, implementing regular supportive supervision for TB facilities, adopting drug adherence strategies to enhance TB treatment outcomes, implementing interventions to reduce treatment defaults, providing patient counseling, and giving more attention to vulnerable age groups and HIV-positive individuals.

## Supporting information

S1 Dataset (XLSX)
